# Vitamin D Deficiency in End Stage Renal Disease Patients with Diabetes Mellitus Undergoing Hemodialysis

**DOI:** 10.7759/cureus.11668

**Published:** 2020-11-23

**Authors:** Muhammad Ali, Ayesha Ejaz, Shafique A Solangi, Abdul Manan Junejo, Mahjabeen Yaseen, Hina Iram, Sagheer Ahmed Solangi

**Affiliations:** 1 Nephrology, Fazaia Ruth Pfau Medical College, Karachi, PAK; 2 Nephrology, Jinnah Postgraduate Medical Centre, Karachi, PAK; 3 Medicine, Jinnah Postgraduate Medical Centre, Karachi, PAK

**Keywords:** chronic kidney disease, diabetes mellitus type 2, hypovitaminosis, vitamin d

## Abstract

Objectives: To assess the association of hypovitaminosis D with diabetes mellitus (DM) in patients with end stage renal disease (ESRD) undergoing hemodialysis.

Methodology: This cross-sectional study was conducted at the Jinnah Postgraduate Medical Centre between July 2019 and February 2020. Patients with diagnosed ESRD who were on hemodialysis, with or without concomitant DM were registered. Vitamin D levels were categorized according to the severity of the deficiency or excess as 0-10 ng/mL, severely deficient; 11-20 ng/mL, deficient; 21-32 ng/mL; insufficient, 33-49 ng/mL, adequate; 50-65 ng/mL, optimum; and above that as high. Patients were stratified according to the status of DM.

Results: In a total of 80, the mean age was 45.21±12.67 years with 51 (63.75%) males and 29 (36.25%) females. A total of 36 (45%) CKD patients had concomitant diabetes. The median vitamin D levels were 20.25ng/mL. It was found that chronic kidney disease (CKD) patients with concomitant DM had significantly lower levels of vitamin D [15.19±6.83 vs. 30.28±14.22 (p<0.001)]. Out of the 12 patients with a severe deficiency, three-fourths of the population had DM as comorbidity, while in those with ‘deficiency’, 19 (67.9%) had DM. The majority of the patients without DM had adequate or optimum levels of serum 25-hydroxyvitamin D levels.

Conclusion: Current study indicated that deficiency of serum vitamin D is associated with concomitant DM in patients with CKD as the majority had a severe deficiency of serum 25(OH)D. Supplemental vitamin D may help correct the deficiency and prevent the associated complications in patients.

## Introduction

Vitamin D is a fat-soluble vitamin that is produced endogenously in the skin depending on the ultraviolet rays from the sun, which when in contact with the skin triggers vitamin D synthesis in the body. For most individuals, around 90% of vitamin D is produced in this way, whereas the remaining 10% is obtained from food and dietary supplements [1-2].

Previously, it was known that this vitamin only plays a role in the regulation of calcium and phosphate in our body [2]. However, more recently low levels of vitamin D have also been linked with many other conditions including bone diseases, cardiovascular diseases, and many psychiatric ailments [3-5].

Provitamin from the skin or diet after hydroxylation in the liver forms 25-hydroxyvitamin D [25(OH)D]. It is converted into its activated form (1,25-dihydroxyvitamin D) in the kidneys [6]. In chronic kidney disease (CKD) patients, this final step of active vitamin D production is impaired. Moreover, the hyperphosphatemic-osteocyte-derived hormone, i.e. fibroblast growth factor (FGF-23) increases to compensate for phosphate retention and further inhibits the renal 1α-hydroxylase expression, inducing the expression of 24-hydroxylase responsible for the degradation of 1,25-dihydroxyvitamin D3. However, 24,25-dihydroxyvitamin D3 levels are lower in dialysis patients than in the normal population. Thus, the impaired uptake of 25-hydroxyvitamin D3 by altered kidneys remains the main cause of 1,25-dihydroxyvitamin D3 deficiency [7].

Another important clinically significant discussion could be of the substrate deficit, i.e. decreased 25(OH)D. Patients with CKD or those on hemodialysis are believed to have reduced cutaneous synthesis than normal individuals as well as increased melanin pigmentation, even if the sunlight exposure is identical. This might be the reason for the low levels of 25(OH)D. Other reasons may be inactivity (low exposure) or inadequate calcium containing diet. Interestingly, they have a direct relationship between 1,25 dihydroxyvitamin D and 25(OH)D unlike normal individuals, the exact cause of which is unknown [8,9].

Evidence has shown that increasing age, female gender, proteinuria, physical inactivity, diabetes mellitus (DM), and nutritional deficiencies are correlated with hypovitaminosis D in patients with CKD [5,6]. Nephropathy is a serious complication that develops in patients with DM. Diabetic kidney disease affects about one-third of patients with DM and currently ranks as the foremost cause of end-stage renal disease (ESRD) [10]. Low levels of vitamin D have been reported in CKD patients with concomitant DM undergoing hemodialysis therapy [5]. Furthermore, Drechsler et al. reported that deficiency of vitamin D in patients on hemodialysis leads to adverse outcomes and is associated with high mortality among these patients [6].

Despite a high prevalence of vitamin D deficiency as well as DM, especially in Pakistan the local data has been very limited [11,12]. Hence, the current study was undertaken to fill the gap in the local literature. The current study aimed to determine the association between vitamin D deficiencies in CKD patients with DM undergoing hemodialysis compared to non-diabetic patients in our setting. 

## Materials and methods

This cross-sectional observational study was conducted at the Nephrology Department, Jinnah Postgraduate Medical Centre, Karachi, Pakistan between July 2019 and February 2020. Ethical approval was procured from its Institutional Review Board. A non-probability convenience sampling technique was applied and 80 patients with diagnosed ESRD who were on hemodialysis, with or without concomitant DM were registered in the specified duration. All patients were included in the study after informed verbal and written consent.

According to the World Health Organization, vitamin D deficiency is defined as the serum 25(OH) D levels of less than 20 ng/mL [13]. For this study, vitamin D levels were categorized according to the severity of the deficiency or excess among the participants. It was grouped into seven classes as: severely deficient 0-10ng/mL, deficient 11-20 ng/mL, and insufficient 21-32ng/mL, adequate 33-49 ng/mL, optimum 50-60 ng/mL, toxic 60 -70ng/mL and above that as potentially toxic [14].

For blood samples, an experienced nurse was appointed who had experience of more than three years. A 5 ml blood sample was collected from each patient, under aseptic conditions using a tourniquet. Patients' vitals were monitored regularly. Serum samples were collected for hemoglobin (Hb) levels, serum vitamin D, intact parathyroid hormone (iPTH), urea and creatinine, hemoglobin A1c (HbA1c), ferritin, transferrin, random blood sugar (RBS), and fasting blood sugar (FBS). Patients were stratified according to the status of DM diagnosed by RBS, FBS, and HbA1c. For all participants, demographic data and clinical characteristics were entered into a predefined questionnaire.

Data were analyzed using IBM Statistical Package for the Social Sciences (SPSS) Statistics for Windows, version 21.0. (IBM Corp., Armonk, NY, US). The average age, Hb, vitamin D, iPTH, among other continuous variables were presented as mean and standard deviation. For vitamin D levels, the median was also presented. Categorical data were represented as frequency and percentages. Association of vitamin D levels with DM status was observed using a Chi-square test and an independent t-test. A p-value <0.05 was set as the cut-off value for significance.

## Results

In a total of 80 participants, the mean age was 45.21 ± 12.67 years with a range of 18-40 years. There were 51 (63.75%) male and 29 (36.25%) female participants. The mean values for over all serum Hb, iPTH, vitamin D levels, calcium and phosphate are given in Table 1.

**Table 1:** Clinical Characteristic of Patients in the Study Population Abbreviations: SD: Standard deviation; URR: Urea reduction ratio; SpKt/V: Single pool Kt/V where Kt/V shows dialysis adequacy by incorporating dialyzer clearance of urea (K), dialysis time (t), and volume of distribution of urea (V); iPTH: Intact Parathyroid hormone

Variable	Mean ± Standard Deviation
Age ± SD in years	45.21 ± 12.67
Hemoglobin ± SD in mg/dL	9.81 ± 1.7
iPTH ± SD in pg/dL	592.4 ± 624.78
Vitamin D ± SD in ng/dL	23.5 ± 13.70
Serum ferritin ± SD in pg/dL	560.27 ± 371.49
Iron ± SD in mcg/dL	91.50 ± 41.61
Calcium ± SD in mg/dL	8.11 ± 0.824
Phosphate ± SD in mg/dL	5.673 ± 1.705
Albumin ± SD in mg/dL	3.33 ± 0.538
URR ± SD in percent	64.074 ± 4.404
SpKt/V	1.225 ± 0.115
Dialysis Duration ± years	5.212 ± 1.004
Dialysis sessions-week	2.606 ± 0.5126

We have stratified patients, a total of 36 (45%) ESRD patients had concomitant diabetes while 44 (55%) did not have diabetes as a comorbidity. The overall mean vitamin D levels were 23.5 ± 13.70 with a range of 4.82-68.90 ng/dL. It was found that ESRD patients with concomitant DM had significantly lower levels of vitamin D compared to patients without diabetes as comorbidity [20.517 ± 12.447 vs. 30.021 ± 12.810 (p<0.0004)]. Out of the 12 patients with a severe deficiency, three-fourths of the population had DM as comorbidity. Similarly, out of the 28 patients with deficient levels, 19 (67.9%) had DM (Table 2).

**Table 2:** Comparison of parameters between diabetic and non-diabetic patients Abbreviations: URR: Urea reduction ratio; SpKt/V: Single pool Kt/V where Kt/V shows dialysis adequacy by incorporating dialyzer clearance of urea (K), dialysis time (t), and volume of distribution of urea (V); PTH: Parathyroid hormone

n=94		Diabetic n= 45	Non Diabetic n=49
Variable	P= value	Mean ± Standard Deviation	Mean ± Standard Deviation
Age	0.577	46.31 ± 13.534	44.78 ± 13.039
Uric Acid	0.222	6.980 ± 2.069	6.3790 ± 1.909
Hemoglobin	0.861	9.2342 ± 1.329	9.285 ± 1.516
PTH	0.721	563.133 ± 580.703	606.101 ± 585.320
Iron	0.948	93.952 ± 49.391	93.343 ± 39.910
Feritin	0.242	641.2267 ± 429.775	548.574 ± 368.994
Calcium	0.755	8.1398 ± 0.855	8.0861± 0.802
Phosphate	0.437	5.687 ± 1.477	5.6602 ± 1.906
Albumin	0.885	3.347 ± 0 .495	3.3310 ±0.580
URR	0.753	64.93 ± 4.002	64.67 ± 3.996
SpKt/V	0.816	1.1489 ± 0.132	1.1429 ± 0.117
Year	0.045	4.422 ± 0.811	4.326 ± 0.875
Sessions /W	0.918	2.622 ± 0.490	2.632 ± 0.487
Vitamin D	0.0004	20.517 ± 12.447	30.021 ± 12.810

In our study adequacy of hemodialysis was also assessed by urea reduction ratio and single pool Kt/V; in the diabetic group mean URR was 64.93 ± 4.002 while in the non-diabetic group it was 64.67 ± 3.996 with a P-value of 0.753.

SpKt/V was 1.1489 ± 0.132 in the diabetic group and in the non-diabetic group it was 1.1429 ± 0.117 (P-value 0.816).

Mean duration of hemodialysis patients was 5.212 ± 1.004 years, while in the diabetic group duration of hemodialysis 4.422 ± 0.811 years and in the non-diabetic group it was noted 4.326 ± 0.875 with significant P-value (0.045).

**Figure 1:**
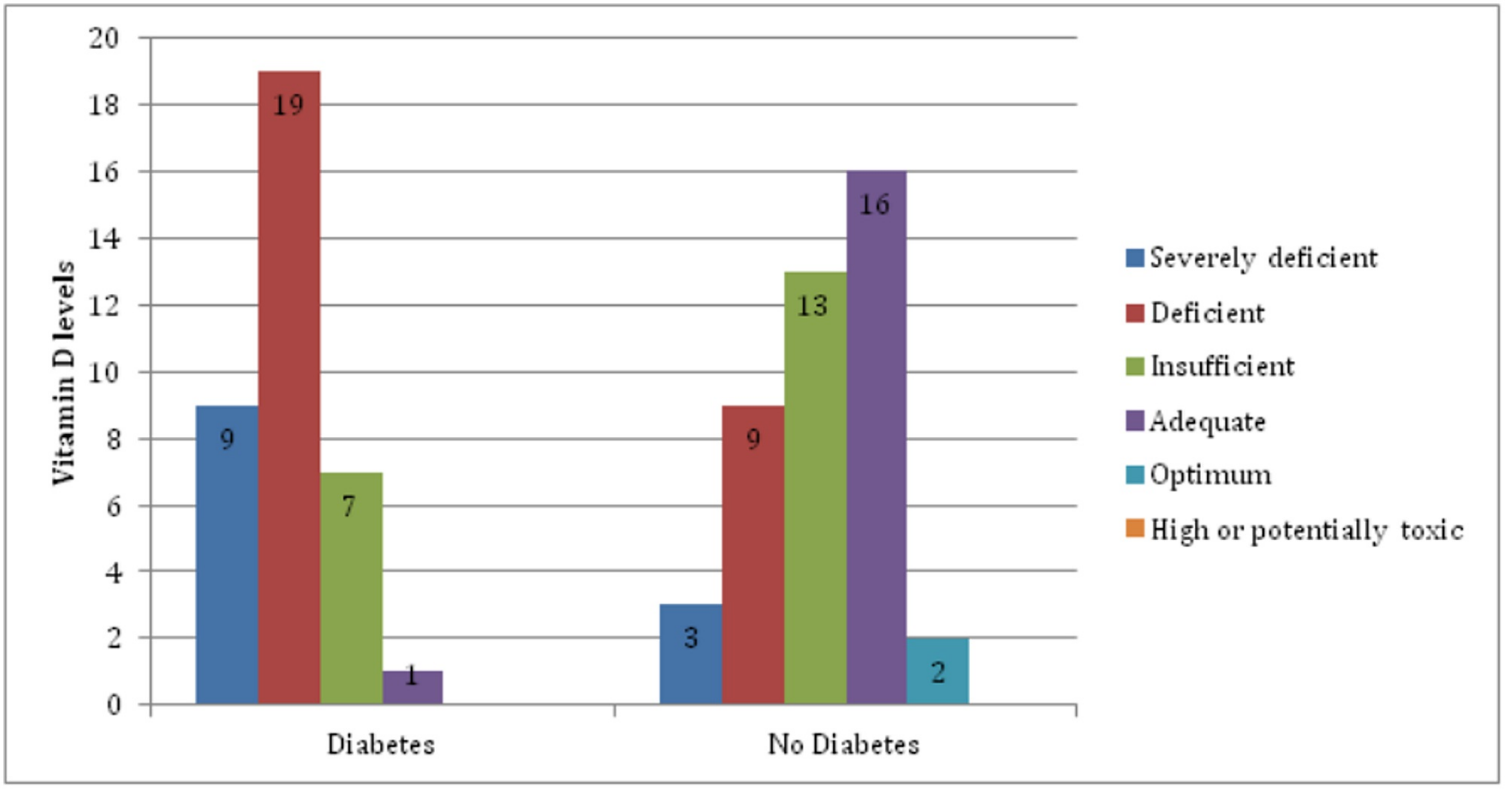
Association of diabetes mellitus with vitamin D deficiency among patients with end-stage renal disease

The majority of patients without diabetes mellitus had adequate or optimum levels of serum 25-hydroxyvitamin D levels (Figure 1).

## Discussion

The degree of association of vitamin D, serum mineral regulation, and the physiological function of the renal system necessitate a vast body of research that may provide relevant clinical insight. In this study, the mean values for Hb, iPTH, and vitamin D were decreased, increased, and normal respectively following standard ranges. These findings demonstrate that overall vitamin D deficiency was not particularly prevalent in this population of ESRD patients; although these levels did not reflect a deficiency, they are still relatively low within the normal range.

Even though vitamin D deficiency was not demonstrated based on mean serum levels within the population, this study was able to find a statistically significant difference between vitamin D serum levels relative to non-diabetic ESRD patients. As stated earlier in the results, out of the patients with the greatest level of vitamin D deficiency, the vast majority of them had DM as associated comorbidity. Other studies have demonstrated a linear progressive relationship between diabetic nephropathy patients and vitamin D deficiency [15]. Sacerdote et al. performed a focused review and found an inverse relationship between vitamin D levels and type 2 diabetes, as well as other insulin-related disorders [16].

The causal mechanism involved in the onset and progression of diabetes in association with vitamin D likely concerns the role of the vitamin in the immune system and insulin secretion. For instance, vitamin D serves an important immunomodulatory function, and its respective receptor (VDR) is found on both T and B lymphocytes. The modification of the T helper cell cytokine profile can induce inhibition and expression of effectors T cells, such as those involved in autoimmune reactions which may lead to type 1 DM, vitamin D deficiency also inhibits pancreatic insulin secretion from the beta islet cells, contributing to complications observed in type 2 diabetes [17]. The clinical implications of these findings may be especially immense given local studies that found statistical significance among vitamin-deficient patients and elevated HbA1C levels, blood glucose, and poor glycemic control [18]. Mahmood et al. concluded that measures should be taken to avoid unfavorable clinical outcomes in DM patients through either vitamin supplementation or increased exposure to sunlight [19].

Despite efforts to generalize the findings, this study still posed several limitations such as limited sample size and a primarily older, male, and anemic demographic. The clinical profile and demographics of the patients provide preconditions that are inextricably linked to the propensity for hypovitaminosis. It is well known that the process of aging affects the formation of vitamin D. In fact, an approximately 50% reduction of production is observed due to a decline in renal function which is largely caused by progressing age. The development of a deficiency leads to a greater reduction in the formation of the metabolite, initiating a sort of positive feedback loop. Besides, gender has also been found to play a largely significant role in vitamin D status. In a study evaluating patients undergoing coronary angiography, females had higher rates of renal failure and were associated with lower vitamin D levels by a large statistical margin. Furthermore, recent studies have found that vitamin D deficiency tends to coincide with anemia in both healthy and diseased populations, and may even be involved in the causal mechanism of its development. Granted its involvement in erythropoiesis and the suppression of hepcidin, there is evidence that sufficient supplementation of the vitamin may be an effective preventative measure against the development of anemia [20-22].

There is significant relationship between duration of hemodialysis and vitamin D levels in diabetic patients on hemodialysis (0.045), while Bansal et al. in 2012 from India found weak correlation between duration and vitamin D [23]. El‑Arbagy et al. from Egypt recently stated that there is no correlation between duration and vitamin D level [24].

## Conclusions

The current study indicated that deficiency of serum vitamin D is associated with concomitant DM in patients with ESRD as the majority had a severe deficiency of serum 25(OH)D. Supplemental vitamin D may help correct the deficiency and prevent the associated complications in patients.
